# Research on Discrete Semantics in Continuous Hand Joint Movement Based on Perception and Expression

**DOI:** 10.3390/s21113735

**Published:** 2021-05-27

**Authors:** Lesong Jia, Xiaozhou Zhou, Hao Qin, Ruidong Bai, Liuqing Wang, Chengqi Xue

**Affiliations:** School of Mechanical Engineering, Southeast University, Nanjing 211189, China; lesong@seu.edu.cn (L.J.); 220184307@seu.edu.cn (H.Q.); 220200377@seu.edu.cn (R.B.); 220194557@seu.edu.cn (L.W.); ipd_xcq@seu.edu.cn (C.X.)

**Keywords:** hand gesture, semantic cognition, discreteness of cognition, human mirroring mechanism, Leap Motion

## Abstract

Continuous movements of the hand contain discrete expressions of meaning, forming a variety of semantic gestures. For example, it is generally considered that the bending of the finger includes three semantic states of bending, half bending, and straightening. However, there is still no research on the number of semantic states that can be conveyed by each movement primitive of the hand, especially the interval of each semantic state and the representative movement angle. To clarify these issues, we conducted experiments of perception and expression. Experiments 1 and 2 focused on perceivable semantic levels and boundaries of different motion primitive units from the perspective of visual semantic perception. Experiment 3 verified and optimized the segmentation results obtained above and further determined the typical motion values of each semantic state. Furthermore, in Experiment 4, the empirical application of the above semantic state segmentation was illustrated by using Leap Motion as an example. We ended up with the discrete gesture semantic expression space both in the real world and Leap Motion Digital World, containing the clearly defined number of semantic states of each hand motion primitive unit and boundaries and typical motion angle values of each state. Construction of this quantitative semantic expression will play a role in guiding and advancing research in the fields of gesture coding, gesture recognition, and gesture design.

## 1. Introduction

As a natural form of semantic expression, gesture occupies an important position in the field of multimodal interaction [1,2,3]. Due to the limited range of hand joint motion and the discrete perception of the state, the continuous movement of a hand motion primitive unit only contains a limited number of semantic states. This article hopes to explore the discrete space of the semantic state of gestures by integrating perception and expression, and to provide convenience for natural interactive gesture coding and gesture design through specific and quantified classification results. To this end, this section separately introduces the movement and restraints of hand joints, the perception and expression of gesture semantics, the research status and limitations of discrete expression space of gesture, and the research content of this article.

### 1.1. Movement and Restraints of Hand Joints

**Figure 1:**
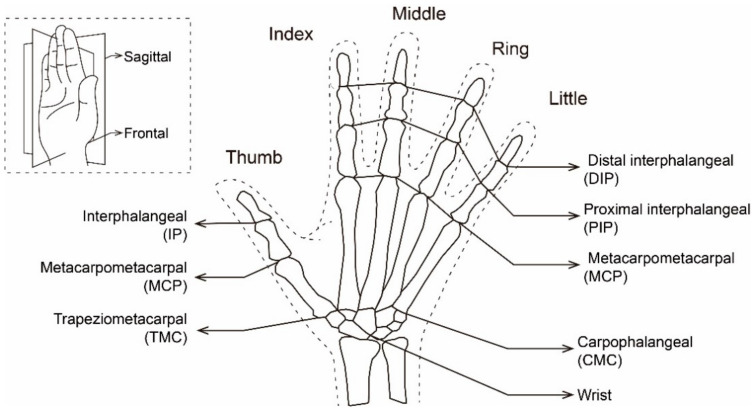
Structure and naming method of hand skeleton.

The motion angles of each joint of the hand and wrist together constitute the gesture posture, which conveys different semantics. Due to the different anatomical structure and movement methods, studies of sign language typically discuss the thumb separately from the other four fingers, which are usually discussed together [4,5,6]. Figure 1 shows the general hand skeleton structure and the corresponding naming method. The joints that control the thumb movement are called trapeziometacarpal (TMC), metacarpophalangeal (MCP), and interphalangeal (IP). The control joints corresponding to the other four fingers are named carpometacarpal (CMC), metacarpophalangeal (MCP), proximal interphalangeal (PIP), and distal interphalangeal (DIP) [7,8,9].

There is a large volume of research on the range of joint motion, including active range of motion (aROM) [10,11,12,13,14,15] and functional range of motion (fROM) which represent activities of daily living [7,11,12,15,16]. Depending on the joints and movement, fROM is 5° to 28° smaller than the available aROM [11,12,16]. The joint motion range corresponding to the execution process of the intentional gesture should be aROM. The aROM of the wrist and hand joints in the two orthogonal directions reported by the relevant research is shown in Table 1.

**Figure 2:**
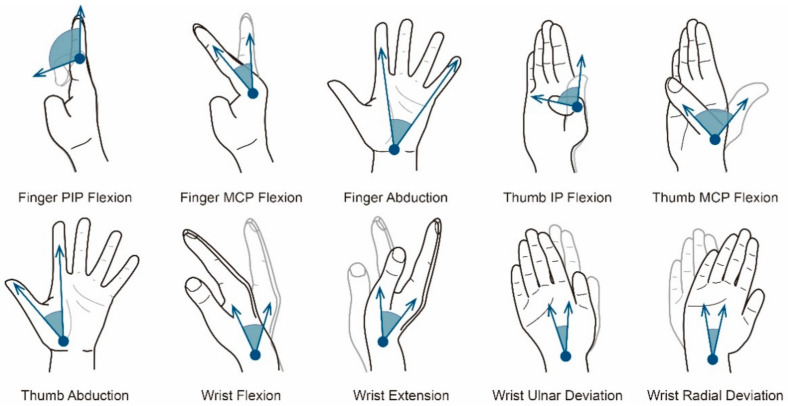
The motion forms of motion primitive units.

In addition, many studies have shown that there are certain constraints between the movement of different joints of the hand. In the study of gesture sign language, the constraints that are widely accepted and recognized by scholars include the finger DIP flexion following the finger PIP flexion, and the thumb TMC flexion following the thumb MCP flexion [6,17,18,19]. Therefore, as shown in Figure 2, for the clear of a gesture, it is necessary to determine the finger MCP flexion, finger PIP flexion, figure abduction, thumb MCP flexion, thumb IP flexion, thumb abduction, wrist flexion, wrist extension, wrist radial deviation, and wrist ulnar deviation. The above motion forms of joints could be called motion primitive units, which constitute the basic movement elements of gestures and are our research object.

In studies of motion angles of the joints of wrist and hand, predecessors mainly focused on the detection of the range of motion function and the exploration of the constraint relationship of the hand-joint motion. However, their discussion did not combine the semantic state perception of the hand with the angle of joint motion. This makes the semantic state of gestures unable to be quantitatively described and accurately classified. Moreover, this further affects the practicality of discrete semantic states in gesture design, recognition, and generation.

### 1.2. Perception and Application of Gesture Semantics

Hand movement is an external representation of states. However, the brain can assign meaning to a hand motion to give it internal meaning, and the motion could thus be regarded as a gesture. In the operational context, the human mirror mechanism allows for the transition from ‘doing something’ to ‘transmitting it to others’ [20]. Neurophysiology research has provided evidence of this consistency. Investigators found the same frontoparietal networks were activated when monkeys [21] or humans [22,23] received the visual input of certain operations through observation, or performed the same action. Therefore, the perception and expression of gestures are consistent [24], which is also the basis for using gestures to convey semantics.

The great complexity and flexibility of human hands means that gestures have a rich coding space and can transmit a variety of semantic information. As a general symbolic expression channel, gestures represent an approach to social communication and an essential component of natural human–computer interaction. With the popularization of gesture recognition equipment and the development of gesture recognition technology, gesture and gesture interaction have been widely used in many fields [25], such as robot control [1], non-verbal communication [26], virtual reality [27], home automation [28], and medical applications [29].

### 1.3. Discreteness of Gesture Semantic Perception

From the perspective of human cognition, although the external information changes continuously, the transformation of meaning is discretely distributed. Long-lasting postdictive effects proved that the meaning transformation in human brains occurs in discrete trajectories, and the boundaries of the trajectory are divided by first-order state conversion [30]. For example, people use the octave with an increasing ratio to characterize the level of the scale. Many studies have demonstrated that humans have a limited number of perceptual orders for the continuously variable stimuli from the external environment [31], and people have absolute recognition ability in different stimulus dimensions [32]. For example, the number of color wavelengths that humans can distinguish is nine, and the number of identifiable vibration intensities is five. In summary, it could be argued that people’s perception of the meaning of external continuous stimuli is limited and discrete.

Human expression of gestures also follows the above rules. In theory, the continuous movement of the hand joints can produce an infinite number of gestures. However, the physical constraints and intentional semantic expression of human hands make effective gestures limited and discrete. Clear and objective discrete gesture expression conforms to human cognition and expression and facilitates the communication and exchange of gesture meaning between people, especially professional gesture researchers and designers. Such clear expression also facilitates the conversion of gesture intentions into discretized computer storage strings, which are convenient for computer storage and interpretation, and for further use for the communication, recognition, and design of gestures.

### 1.4. Research Status and Limitations of Discrete Expression Space of Gesture

At present, the research on the discrete expression space of gestures mostly focuses on the coding of gestures. In the existing gesture coding system, the idea of discretization has been frequently adopted. For example, in the semantics-based sign-language systems, the widely recognized symbol systems include HamNoSys [33], Stokoe Notation [34], and SignWriting [35], all of which discretize and encode gestures. However, the existing gesture coding strategies—for instance the widely used HamNoSys coding scheme [36]—have merely classified semantic state representations qualitatively (e.g., curved, semi-curved, and non-curved), without determining the specific angle threshold and characteristic value of joint motions corresponding to each semantic state. Similarly, Stokoe Notation and SignWriting use characters and images to represent the different motion states of finger bending and finger abduction [34,35]. However, these states are still only a vague concept, and there is no specific motion value reference. In addition, these classifications of semantic states were summarized specifically for sign-language gestures, lacking integration with the theory about human cognition of gesture semantics. Such classification could be affected by the subjective bias of coders; consequently, it may not be able to express people’s real understanding of the semantic state of gestures.

### 1.5. This Study

Research on gestures provides practical discrete gesture codes [33,34,35] and the range of motion and constraint relations of each joint of the hand [6,11,17,18,19,37]. However, the discrete classification of the motion state of each joint still lacks objective consideration from perception and expression aspects and has not been correlated with the motion angle of joints to give a clear angle threshold and characteristic value. Besides, the measurement deviation of the hand gesture capture device represented by Leap Motion also leads to the inconsistent between actual intention and the recognition intention in the gesture interaction. These are the problems that the current study set out to explore and solve.

The present article examines the meaningful angle classification of the movement of hand motion primitive units both in the real world and Leap Motion digital world, using four experiments. Experiments 1 and 2 used the constant stimulus method from psychophysics to determine the number of perceivable states of different angle states of motion primitive units and the corresponding motion angle range of each state from the perspective of meaning perception. Experiment 3 was based on the human mirror mechanism. From the perspective of gesture expression, the participants were asked to divide the motion expression state of the motion primitive units and make a typical gesture for each state to verify the reliability and validity of the angle threshold, optimize and iterate the angle range, and further clarify the characteristic angle value of each state. Based on the above, in Experiment 4, the adjustment proposal for the gesture capture algorithm of Leap Motion was given by analyzing the measurement error. Furthermore, the angle boundary values of the motion states of each motion primitive unit based on Leap Motion were given for use at current stage by mapping between the actual and measured values of Leap Motion.

## 2. Methods

**Figure 3:**
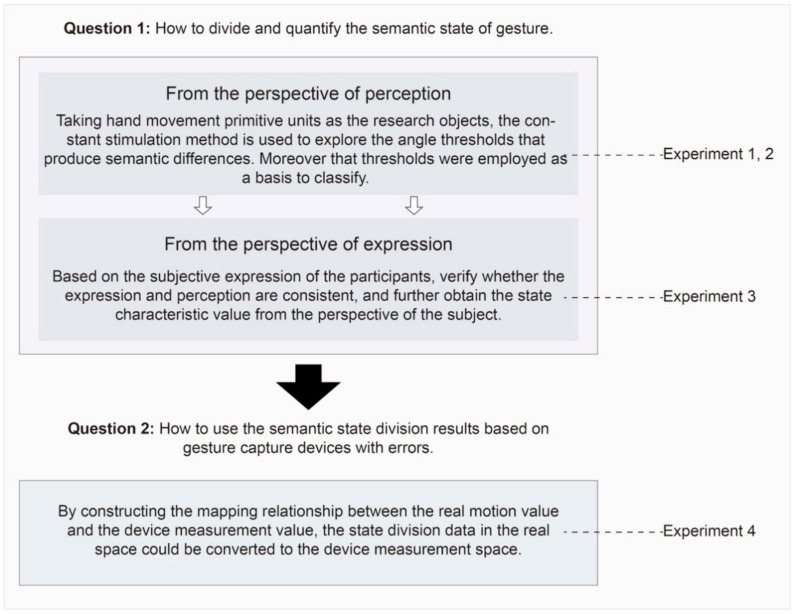
Experimental design framework diagram.

As shown in Figure 3, Experiments 1, 2, and 3 were designed to obtain discrete gesture semantic space, including the number of semantic states of each motion primitive unit and the feature and critical value of each state. Moreover, a further experiment, i.e., Experiment 4, was designed, taking Leap Motion as an example to verify the practical application of gesture semantic state division. This section introduces the methods from three aspects: semantic perception state division based on constant stimulus method, state classification evaluation and feature angle extraction based on subjective expression, and practical example of semantic state division based on Leap Motion and data mapping.

### 2.1. Semantic Perception State Division Based on Constant Stimulus Method

To perceptually obtain the semantic state division of each motion primitive, Experiments 1 and 2 are designed to explore the number of semantic states that each motion primitive can convey and the motion angle boundary of each state based on the constant stimulus method.

The method of constant stimuli (also known as the method of right and wrong cases or frequency method) was one of three classic psychophysical methods for studying sensory absolute and differential thresholds [38]. This method is widely used in related fields [39,40,41]. For the measurement of the difference threshold, a typical experimental paradigm of the constant stimulation method is repeatedly presenting a series of contrast stimuli in random order and asking the observer to report on whether there is a difference with standard stimulus. The threshold is determined to be the point at which the subject notes a just noticeable difference from the comparison stimulus on 75% of the trials (average Z score is equal to 0.67).

For our research, pictures of gestures were presented to the subjects as stimuli. Gesture pictures are generated by hand models and renderings to facilitate precise control of the hand’s joint movement angle. Each stimulus picture included a standard gesture and a contrasting gesture. Standard gestures include the extreme state and the central state of the motion primitive unit movement. Contrasting gestures are sequences in which the movement angles are arranged in equal difference to the standard gestures. Participants need to judge whether the semantics of the two gestures in each set of stimuli are consistent. Through the statistical analysis of the judgment data, the semantic difference threshold of each motion primitive unit can be obtained. Furthermore, the semantic state of the hand primitive unit can be divided.

### 2.2. State Classification Evaluation and Feature Angle Extraction Based on Subjective Expression

Through the abovementioned perception experiment, the number of semantic states of each motion primitive unit and the critical angle of each state are clarified. Next, we hope to verify that the subjective expressions are consistent with the perception-based division. In addition, the typical value of the expression of each semantic state also needs to be clarified.

For this reason, Experiment 3 was designed. First, participants were asked to judge the number of semantic states included in each motion primitive unit from the perspective of the gesture performer. Then, the subjects need to execute the representative movement angle corresponding to each state. As a result, the number of states and execution angles could be measured and recorded to evaluate the consistency with the perceptual classification results. Moreover the characteristic values of each state could be given by calculating the average of the execution angles.

### 2.3. Practical Example of Semantic State Division Based on Leap Motion and Data Mapping

Through the above experiments, we finally clarified the semantic expression space in the real world. However, gesture capture devices represented by Leap Motion will produce a certain deviation when capturing gestures. Therefore, to apply semantic classification to these devices, it is necessary to map the angle values of the semantic state division in the real world to the measurement space of these devices.

For that reason, Experiment 4 was designed to demonstrate the mapping method. For each motion primitive, five arithmetic target motion angles are used for calibration. First, the participants need to make gestures according to the target angle with the assistance of a measuring ruler. Then, gesture capture devices were used to measure the movements. Finally, the mapping equation can be constructed through the linear function fitting between the measured value and the real value. As a result, the division angle corresponding to the semantic state can be mapped to the device measurement space.

**Figure 4:**
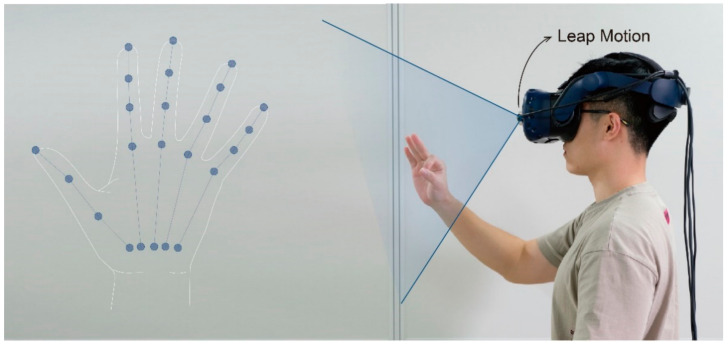
Schematic diagram of Leap Motion hand gesture.

Leap Motion was used as the gesture capture device for Experiment 4. As shown in Figure 4, Leap Motion is the most widely used non-contact hand gesture capture sensor [42,43,44], which can provide the position and motion data of each joint of the hand in desktop mode or VR mode. Based on these data, information such as the bending angle of each joint and the abduction angle of the fingers can be further obtained and used to gesture recognition and gesture interaction. Nevertheless, a certain systematic measurement error about 15 percentage of the hole motion range was demonstrated in the experiments [45]. Therefore, in some cases, the data given by Leap Motion may not truly reflect the user’s hand gestures, resulting in a degradation of the efficiency of Leap Motion–based gesture recognition and the experience of gesture interaction.

## 3. Experiment

The above method section gives the research methods and ideas of this study. This section introduces the specific design, results, and discussion of the four experiments.

### 3.1. Experiment 1: Measurement of Semantic Difference in Visual Perception of Motion Primitive Units

Experiment 1 was carried out to provide preliminary clarification of the number of semantic states of each motion primitive unit and the range of motion angles of each semantic state by measuring the visual semantic perception difference of motion primitive unit. The method of constant stimulus from the field of psychophysics was used. A contrast gesture and a standard gesture were presented in an experimental stimulus at the same time. The standard gestures could also be called boundary gestures as they corresponded to the motion boundary states of each motion primitive unit, while the contrast gesture was the different motion states of the motion primitive unit arranged in an equal difference to the motion angle. Participants needed to judge whether these two gestures were semantically consistent, and the statistical value of the judgement results was used to quantify the difference in visual perception of two motion states of the motion primitive unit.

#### 3.1.1. Participants and Apparatus

A total of 20 participants (10 males and 10 females) aged between 21 and 26 years (M = 23.3, SD = 1.450) were recruited from Southeast University in Nanjing, China for this experiment. Before the experiment, the participants all read the experimental instructions and gave their written informed consent.

We conducted this experiment in a usability laboratory with good light and a quiet environment. During the experiment, the illumination brightness of the surrounding environment was about 520 lx, and the noise was kept below 30 decibels. The program used for this experiment was developed based on the Unity engine and ran on a PC with a Windows 7 system. The PC was equipped with an NVIDIA GTX 1060 GPU and an Intel Core i7 CPU to support the smooth running of the experimental program.

#### 3.1.2. Task, Procedure and Experimental Design

**Figure 5:**
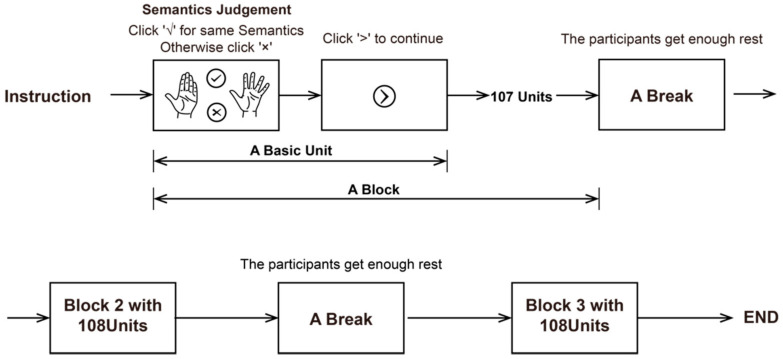
Experimental flowchart of visual perception measurement of semantic differences in motion primitive unit. A total of three blocks are included in the experiment; a block of the experiment consisted of 108 groups of basic units with different stimuli but the same task.

The experiment consisted of multiple groups of experimental units with different stimuli but the same task. The process of each experimental unit is shown in Figure 5.

First, the participants were presented with a randomly ordered set of stimulus gestures. Each stimulus included a contrast gesture and a boundary gesture presented at the same time. The participants were asked to judge whether the two gestures were semantically consistent, not whether the two gestures were exactly the same in appearance. Once the participants completed the judgement, they were asked to click on the check mark to indicate that the two gestures had the same semantics, or on the X mark to indicate that the semantics of the two gestures were inconsistent. The influence of the sequence effect was avoided by randomly swapping the positions of the check and X marks.

Then, during the judgement process, to make the observation of the stimulus more realistic and comprehensive, the participants were allowed to rotate the two gesture models at the same time by clicking and dragging the mouse pointer. Therefore, two gesture models could be observed and compared from all angles to make the observation of stimuli more realistic. In addition, the default gesture presentation angle in the experiment was the angle with the most obvious difference between the boundary gesture and the contrast gesture. At this time, the shooting plane of the virtual camera was parallel to the motion plane of the motion primitive unit, and the participants could more easily identify the motion primitive unit corresponding to the current stimulus. After the judgement of one trial was completed, the participant could click the ‘next’ button that popped up to enter the next trial.

**Figure 6:**
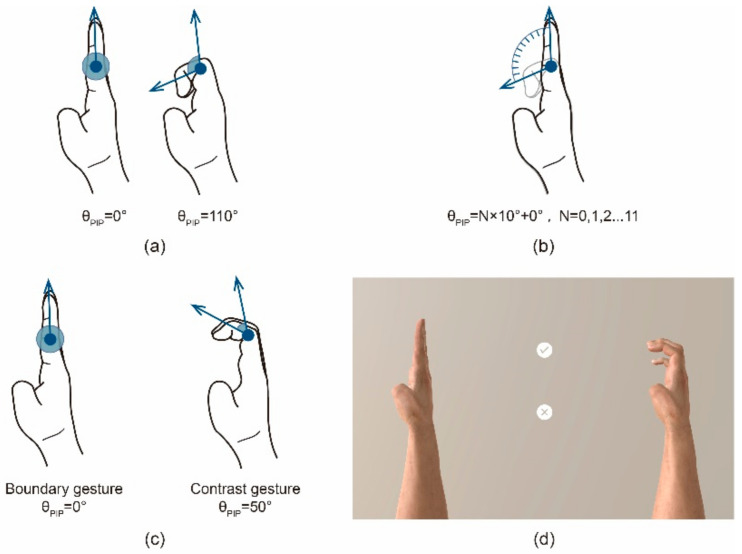
Examples of experimental stimulus selection and presentation: (**a**) the boundary gesture setting, (**b**) the contrast gesture setting, (**c**) an example of stimulus setting, and (**d**) a rendering of (**c**) presented in the experiment.

In the experiment, each stimulus corresponded to only one motion primitive unit, and the motion states of the other joints were consistent for the contrast gesture and the boundary gesture. Furthermore, corresponding to a motion primitive unit, the joint motion angles of the boundary gesture were the maximum and minimum motion angles of the specific motion primitive unit, and the joint motion angles of the contrast gesture were the arithmetic sequence between the maximum and minimum motion angles of the motion primitive unit. Taking the FPIP as an example, the boundary gesture is shown in Figure 6a, and the flexion angles of the corresponding finger PIP are 0° and 110°. Moreover, as shown in Figure 6b, from 0° to 110°, taking a value for the flexion angle of the PIP every 10°, a total of 12 angle values correspond to the contrast gestures.

The configuration of a set of stimuli is shown in Figure 6c. To accurately control the motion angle of each joint, a three-dimensional hand model was used as the carrier of stimulation presentation. A virtual scene was built by the Unity engine, and an HDRP skybox provided a real lighting environment for the scene. The state of each joint of the hand model in the scene could be accurately controlled through data. Charts of the hand model were taken by the virtual camera and presented on the screen. The final rendered image presented to the participants is shown in Figure 6d. The above scheme not only accurately presented the hand posture according to the requirements of the experiment, but also gave the participants the experience of observing the real hand movements.

Table 2 shows the motion form and range of each hand motion primitive unit, as well as the boundary gesture parameters and contrast gesture parameters. Each motion primitive unit was composed of two boundary gestures and several contrast gestures, which formed a total of 162 sets of stimuli. Each set of contrast stimuli would be presented twice, but the positions of the contrast gesture and the boundary gesture were interchanged to reduce the influence of the left and right positions of the gesture on the judgement. Therefore, a total of 162 × 2 = 324 sets of stimuli were included in the experiment, and all stimuli were presented to the participants randomly and without repetition. The experiment was divided into 3 blocks, and each block contained 108 trials. The participants were allowed to rest between the two blocks until they felt well and were ready to continue the experiment.

#### 3.1.3. Results

The judgement results of 20 participants about whether the stimuli are semantically consistent were collected in Experiment 1. The statistical analysis indicated the degree of difference in visual perception for each stimulus, that is, the proportion of the total number of participants who judged that the stimulus had semantic differences.

**Figure 7:**
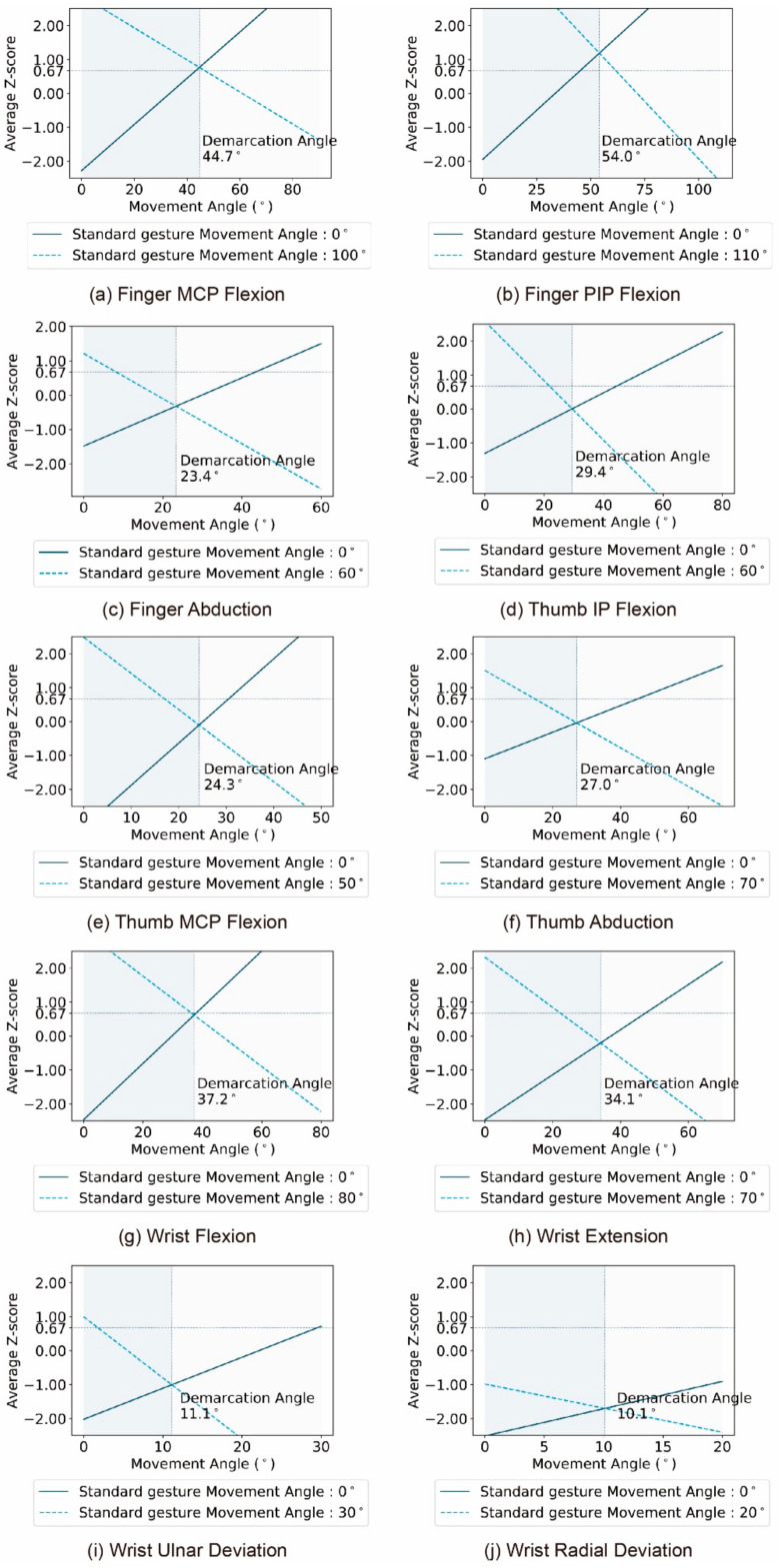
The visual semantic difference perception change curve of the movement primitive unit under different boundary gestures. The two curves in each chart represent the semantic difference changes in visual perception when the contrast gesture was compared with each of the two boundary gestures. The reference line with the ordinate of 0.67 is used as the difference boundary. It is generally believed that, when the average Z score exceeds 0.67, the contrast stimulus and the standard stimulus have a perception difference [38].

As a common practice, the least squares method was used to process data [38]. After excluding extreme values with a difference degree of 0 and 1, the difference degree was converted to the average Z score. Then a linear equation based on the least squares method was fitted, in which the motion angle value of the contrast gesture of the special motion primitive unit was used as the independent variable, and the average Z score corresponding to the difference between the boundary gesture and the contrast gesture was used as the dependent variable. Finally, the change curve of the semantic difference of the contrast gesture under different boundary gestures could be obtained, as shown in Figure 7.

#### 3.1.4. Discussion

Figure 7 shows the visual perception change curves of two gesture semantic differences corresponding to each motion primitive. From the perspective of visual perception, the semantic states of each motion primitive unit can be preliminarily determined through the analysis of the curve and its key points.

First, for each motion primitive unit, the highest points of the two difference perception change curves represent the average Z score of the difference degree between the two-boundary gesture. It can be seen that except for the curve corresponding to the wrist radial deviation, the maximum points of the two curves corresponding to other motion primitive units were higher than 0.67, which means that the two-boundary gestures of these motion primitive units are different in the visual semantic difference perception. Thus, it can be inferred that these motion primitive units can represent at least two semantic states. However, the two maximum values of the two curves corresponding to the wrist radial deviation were much less than 0.67, which indicates that there is no obvious difference between the two boundary gestures of the wrist radial. So, it can be judged that there is only one kind of semantic expression ability with the wrist radial.

Second, at the intersection of the two curves corresponding to each motion primitive unit, the motion angle value of the contrast gesture and the average Z score corresponding to the two curves were equal. This means that the visual perception semantic difference between the contrast gesture corresponding to the intersection of the two curves and the two boundary gestures is equal. Therefore, the angle value corresponding to the intersection of the two curves can be called the semantic median of the motion primitive unit. The semantic median is used as the dividing line, and the motion state of each motion primitive unit can be divided into two regions as shown in Figure 6.

For the motion primitive units with the extreme values of the two curves greater than 0.67, if the average Z score corresponding to the semantic median value is less than 0.67, then the changes in the motion angle of the motion primitive unit in the two divided regions will not make the gesture different in semantic perception. Therefore, the conclusion can be drawn that the above motion primitive unit includes two semantic states, and the semantic median line can be used as the dividing line between the two. Conversely, if the average Z score corresponding to the semantic median value is greater than 0.67, then the changes in the motion angle of the motion primitive unit in the two divided regions will make the gesture different in semantic perception. For this type of motion primitive unit, such as finger PIP and finger MCP, the number of semantic states that they can convey exceeds two, which should be determined through further experiments.

The semantic status of each motion primitive unit can be obtained based on the above judgement criteria and classification methods. First, the single one semantic state can be represented by the radial curvature of the wrist. Second, the two motion primitive units FMCP and FPIP can convey more than two motion states, which need to be further clarified through experiments. In addition, each of the other motion primitive units can convey two semantic states, and the two semantic states can be separated by the semantic median point.

### 3.2. Experiment 2: Visual Perception Semantic State Measurement Experiment of Finger MCP and Finger Pip

The semantic median value of the two motion primitive units, finger MCP and finger PIP, was obtained in Experiment 1. As their average Z scores corresponding to the semantic median exceed 0.67—the threshold of semantic difference—the number of semantic states that these two motion primitive units can convey exceeds two. In this experiment, the number of semantic states that could be conveyed by the two motion primitive units FMCP and FPIP was further explored by using the gesture corresponding to the semantic median as the standard gesture of visual semantic difference perception measurement experiment. To maintain the same judgement criteria, the same participants and experimental equipment from Experiment 1 were used again.

#### 3.2.1. Task, Procedure, and Experimental Design

Two motion primitive units, finger MCP and finger PIP, were studied in Experiment 2. The same tasks and procedures as Experiment 1 were performed in Experiment 2. In the experimental stimulus settings, the standard gesture of each motion primitive unit was set as the gesture corresponding to the semantic median. Therefore, the standard gesture could be called the median gesture. The contrast gesture was set to be consistent with Experiment 1. Similar to Experiment 1, each set of contrast stimuli were presented twice, but the positions of the contrast and boundary gestures were swopped to reduce the influence of the left and right positions of the gesture on the judgement. Taken together, a total of 22 × 2 = 44 sets of stimuli were included in the experiment, which were contained in one block. All stimuli were randomly presented to the participants without repetition.

#### 3.2.2. Results

**Figure 8:**
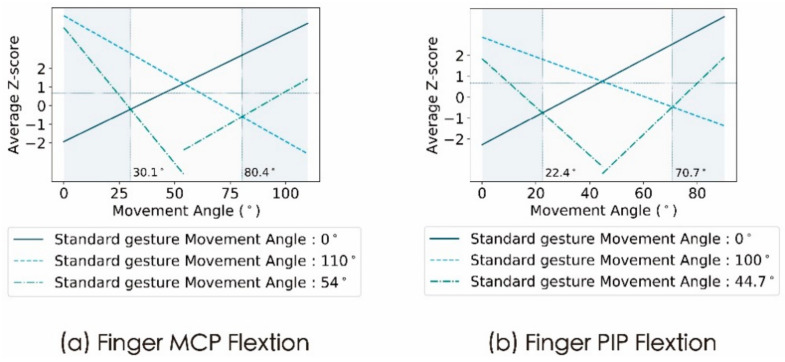
The semantic difference change curve of the contrast gesture under the median and boundary gestures. The reference line with the ordinate of 0.67 is used as the difference boundary. It is generally believed that, when the average Z score exceeds 0.67, the contrast stimulus and the standard stimulus have a perception difference [38]. Moreover, the reference line corresponding to the abscissa marks the intersection between the curves under the median gesture and the curves under the boundary gesture.

The difference degree in visual perception between the contrast gesture and the median gesture of the finger MCP and finger PIP was statistically obtained through Experiment 2. The least squares method was used to analyse and process the data. First, the difference degree was converted to the average Z score, and then the same least squares method as in Experiment 1 was used to perform a linear fit to the average Z score variation curve. As the median gesture was used as the standard gesture, the corresponding change curve of the average Z score of the difference degree in visual perception was fitted in two sections. When the motion angle of the contrast gesture was smaller than the semantic median, the data were fitted as the first part, and when the motion angle of the contrast gesture was greater than the semantic median, the data were fitted as the second part. Combined with Experiment 1, the change curve of the semantic difference of the contrast gesture under the median and boundary gestures could be obtained, as shown in Figure 8.

#### 3.2.3. Discussion

For the finger PIP and the finger MCP, the change curve of the semantic difference of the contrast gesture under the median gestures was drawn on the basis of the original change curve under the boundary gestures as shown in Figure 8. For each chart in the figure, the maximum values of the two sections of semantic difference change curves under the median gestures represent the visual perception between the median gesture and the two boundary gestures. The two maximum values in Figure 8a,b both exceed 0.67. As a result, for the finger PIP and finger MCP, there was a clear difference between the median gesture and the two-boundary gesture. The conclusion from Experiment 1 can be verified, that is, the number of semantic states that can be presented by finger PIP and finger MCP exceed two.

Two intersection points between the two sections of semantic difference change curves under the median gesture and the two curves under the boundary gesture can be seen in Figure 8. These intersection points can be used as demarcation points to divide the entire finger MCP and finger PIP motion range into three parts as shown in Figure 7. Since the average Z score corresponding to the intersection point was less than 0.67, in the left and right parts of the above division, the visual semantic perception of the gesture was consistent with the corresponding boundary gesture, and the visual semantic perception of the gesture in the middle part was consistent with the median gesture. Thus, the number of semantic states that can be conveyed by the two-motion primitive unit finger MCP and finger PIP should be three.

In conclusion, the joint motion of two motion primitive unit finger MCP and finger PIP can be divided into three semantic states and the two intersection points between the semantic difference change curves under the median gesture and the curves under the boundary gesture can be used as the demarcation points.

### 3.3. Experiment 3: Measurement of Semantic States as Expression of Motion Primitive Unit

Based on the mirror mechanism, people’s observation and execution of a hand gesture should be consistent. Therefore, Experiment 3 was designed to verify and optimize the results of Experiments 1 and 2 from the perspective of gesture execution. For the movement primitive units, the number of semantic states and the movement angles of each state were explored from the perspective of the performer. In this experiment, the participants were shown the motion animations of different motion primitive units to help them understand the movement mode of the motion primitive units. Participants were then asked to give the number of states that each motion primitive unit can convey from the perspective of the gesture executor and make typical gestures for each state in turn by themselves. The number of states and the angle of gesture execution recorded in this experiment were used to verify the reliability and validity of the semantic state number and region division obtained through Experiments 1 and 2.

#### 3.3.1. Participants and Apparatus

A total of 20 participants (10 males and 10 females) aged between 19 and 25 years (M = 22.6, SD = 1.560) were recruited from Southeast University for this experiment. Participants in this experiment had not participated in Experiments 1 and 2, to avoid the observation and comparison of stimuli from the observer’s perspective which would interfere with the judgement in this experiment. All participants were right-handed and performed gestures with their right hands to match the right-hand model they were shown. Before the experiment, the participants read the experimental instructions and signed the informed consent form.

**Figure 9:**
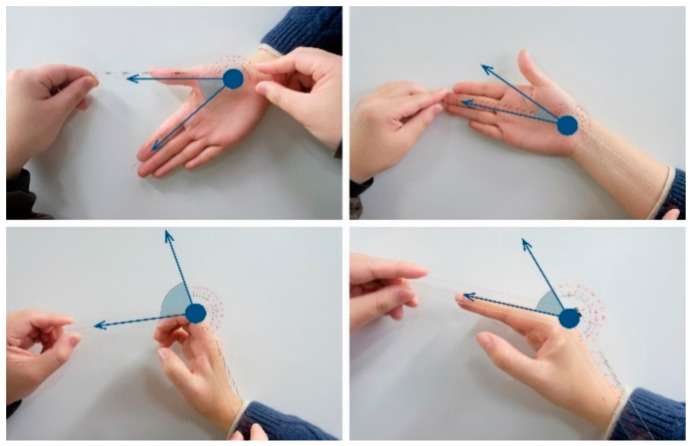
Measurement of the motion angle of the motion primitive units. The blue sector marks the measured angle.

The experimental equipment was consistent with Experiments 1 and 2. The motion animation of the gesture was made in the Unity virtual scene constructed in Experiment 1. Furthermore, as shown in Figure 9, the motion angles of the motion primitive unit were measured by an anthropometer, the universal human-joint measuring equipment [45].

#### 3.3.2. Task, Procedure, and Experimental Design

**Figure 10:**
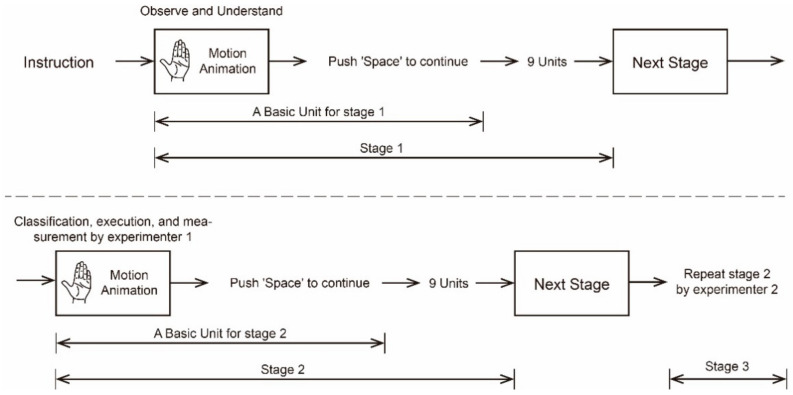
Experimental flowchart of motion primitive unit semantic state expression measurement. The experiment consists of three stages, and each stage has ten basic units, corresponding to ten motion primitive units. The first stage was executed in order to let the subjects understand the motion form of the motion primitive units; the second and third stages used the same process but different experimenters to record and measure the division and execution of the semantic state of each motion primitive units.

As shown in Figure 10, the experiment was divided into three stages. The first stage was implemented to help participants understand the movement patterns of each movement primitive unit. Here, the demonstration animation corresponding to each motion primitive unit was presented to the participants randomly and without repetition. Once the form of movement shown in the current animation was understood by participants, the next animation could be played by pressing the space bar. The first phase of the experiment ended when all the movement modes of the movement primitive units were understood by the participants. In the second stage, the participants were asked to subjectively judge the number of semantic states that each motion primitive unit could convey. Then, for each motion primitive unit, participants were asked to make their own representative gesture for each state. The motion angles of the motion primitive units corresponding to the representative gestures were measured and recorded by Experimenter 1. After that, the tasks and processes of stage two were repeated in stage three, but the data were measured by Experimenter 2. After all the experimental stages were completed, an interview was conducted between the participant and the experimenter to discuss the participant’s thinking during the experiment.

All ten motion primitive units expressed by gestures such as finger MCP flexion, finger PIP flexion, finger abduction, thumb MCP flexion, thumb IP flexion, thumb abduction, wrist flexion, wrist extension, wrist radial deviation, and wrist ulnar deviation were included in this experiment. The demonstration animation corresponding to each motion primitive unit was made on the basis of the virtual scene constructed in Experiment 1. The motion form of the motion primitive unit was presented in the animation to provide vivid explanations and instructions for the participants.

#### 3.3.3. Results

The number of semantic states of each movement primitive unit and the movement angle of the representative gesture corresponding to each state were collected in Experiment 3, from the perspective of the gesture executor. The results are reported as follows.

##### Statistical Analysis of the Number of Semantic Expression States of Motion Primitive Units

**Figure 11:**
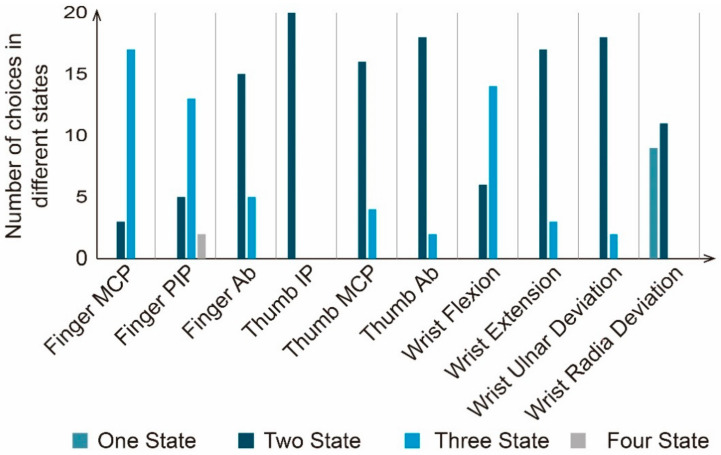
The number of motion primitive unit semantic expression states given by the participants.

Figure 11 presents the classification results for the number of semantic states that each motion primitive unit could convey, given by the participants from the perspective of the performer. The number of semantic states of motion primitive units such as finger MCP flexion, finger PIP flexion, finger abduction, thumb MCP flexion, thumb IP flexion, thumb abduction, wrist flexion, wrist extension, wrist radial deviation, and wrist ulnar deviation not only had high consistency among participants, but were also consistent with the classification results given in Experiments 1 and 2. However, for the semantic classification of wrist flexion, although a high internal consistency can be found between participants, more state numbers were given in Experiment 3 than in Experiments 1 and 2. In addition, a larger divergence arises between the participants regarding the number of semantic expression states of wrist ulnar deviation. Nine of the 20 participants believed that the semantic change would not occur when the wrist is bent on the ulnar side. That is, only one semantic state could be conveyed by wrist ulnar deviation, which was consistent with the conclusions of Experiments 1 and 2. However, the remaining 11 people believed that wrist ulnar deviation could convey two semantic states.

##### Gesture Execution Measurement Results of Each State of the Motion Primitive Unit

For each participant’s subjectively divided semantic state of each motion primitive unit, the execution data of the corresponding typical gestures were measured and recorded. First, for each motion primitive unit, the execution data of the corresponding typical gestures in each state were extracted if the subjective state classification results given by a participant were consistent with the classification results of Experiments 1 and 2. Then, intraclass correlation coefficient (ICC) analysis was performed on the measurement data of each semantic state obtained by the above extraction. ICC is widely used to test the reliability of experimental data and is an index to evaluate the reliability coefficient of inter-observer reliability and test–retest reliability [46]. In the data, the number of semantic expression states of wrist ulnar deviation was 1, and the execution angle of all participants measured by the two experimenters for this state was 0. As the measurement data were completely consistent, the ICC analysis of wrist ulnar deviation measurement data did not provide a usable result. In addition, the data obtained by the two experimenters had extremely high consistency as the ICC values of all other semantic state measurement results were above 0.9 [45].

**Figure 12:**
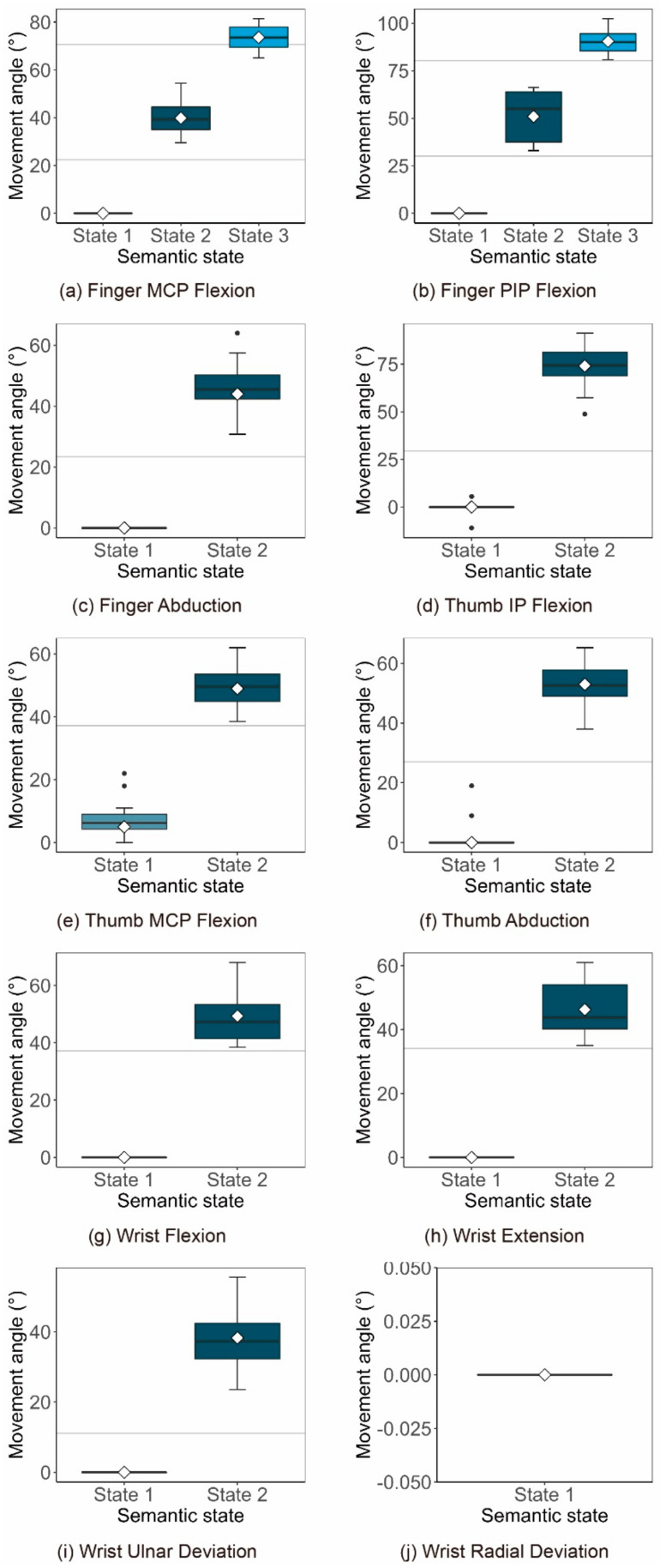
The distribution diagram of the execution angle measurement data of each motion primitive unit semantic state. For each chart, the state name of each motion primitive unit represents the vertical coordinate, while the motion angle of the corresponding motion primitive unit is the horizontal coordinate. Additionally, the horizontal reference lines are the boundary line of the semantic state of each motion primitive unit obtained in Experiments 1 and 2, and the diamonds in the box plot are the mean points of the data corresponding to each state after eliminating outliers.

The subjectivity of the experimenters was further reduced by averaging the measurement results of the two experimenters. Finally, the obtained measurement data of the state of each motion primitive unit were box-plotted as shown in Figure 12. For each chart in Figure 12, the state name of each motion primitive unit represents the vertical coordinate, while the motion angle of the corresponding motion primitive unit is the horizontal coordinate. Additionally, the horizontal reference lines are the boundary line of the semantic state of each motion primitive unit obtained in Experiments 1 and 2, and the diamonds in the box plot are the mean points of the data corresponding to each state after eliminating outliers.

#### 3.3.4. Discussion

Combined with the classification results of the semantic states of motion primitive units obtained in Experiments 1 and 2, the results of Experiment 3 are analyzed in this section from two aspects: the number of semantic expression states and the motion region division of each state.

For the number of semantic states of each motion primitive unit, the results obtained from the expresser and the observer showed a high consistency. Therefore, the rationality of the mirror mechanism of human behavior and the results of Experiments 1 and 2 can be proved to some extent. However, for the wrist flexion and wrist radial deviation, the number of semantic states obtained from the expresser was one more than the number of states obtained from the observer. In the interview with the participants of Experiment 3, some participants said that the sense of exertion produced when the gesture reaches the extreme position is more obvious. Therefore, the states when the motion angle of wrist flexion and wrist radial deviation reaches the maximum value were regarded as a separate semantic state by these participants. This further leads to more semantic expression states from the perspective of the gesture performer. As redundant states are more likely to be caused by invisible rather than explicit motion angle differences, for wrist flexion and wrist radial deviation the number of semantic state classification results obtained in Experiments 1 and 2 are regarded as the final result.

For the motion region division of each state, the consistency shows high retest reliability for the execution of gestures in each state which means that there is a stable and representative motion angle characteristic value corresponding to each motion primitive unit state for the participants. Moreover, the extremely high consistency was shown for the measurement data of the two experimenters, which means that the joint motion angle measurement method used in this article has high inter-observer reliability and is a reasonable and effective method.

As shown in Figure 12, the execution angle values of all states in Experiment 3 were within the area delineated by Experiments 1 and 2, except for the third state of the finger MCP. Hence, for the regional division of the semantic state of each motion primitive unit, the observation and execution of gestures had high consistency. While this further verifies the effectiveness of the human mirror mechanism, the rationality of the semantic state region delineated in Experiments 1 and 2 is also verified. However, in the execution value of the third state of the finger MCP, there are still a few values that exceed the demarcation line.

For the semantic state regional division of finger MCP to be better applied in practice, and to take into account the two categories of performers and observers, the demarcation line between its second and third states can be adjusted appropriately as follows. The adjusted demarcation angle value should be greater than the maximum execution angle of the second state by 49° to avoid additional ambiguity in execution of the second state. In addition, as shown in Figure 12, the new demarcation value also needs to exceed 47° which is the angle value corresponding to the intersection of the semantic difference change curve and the difference boundary line (the average Z score is equal to 0.67). This is so that the semantics of each gesture in the third state area will not be different in visual perception. For the third state of finger MCP, the minimum execution value of the third state of finger MCP obtained in Experiment 3 is 65°. The new demarcation line of this state can be set as 60°, which not only satisfies the above two limiting conditions, but also provides a certain degree of redundancy for the execution of the third state of the finger MCP.

Further, as shown by the diamond mark in Figure 12, after removing the outliers the average value of all the test data in the same state represents the expected value of the motion angle of this state. The average values can be used as the most representative eigenvalue of each semantic state. It can be seen that the characteristic value (38°) of the second state of Wrist Radia Deviation exceeds the maximum value (30°) of the given AROM in Table 1. This might because the maximum angle of motion given by AROM is smaller than the actual average value of the actual to ensure that most healthy people can meet this standard. About the measurement of Wrist Radia Deviation, Kostas Nizamis et al. reported that the average maximum motion angle was 38° [45], and Boone DC et al. reported the average maximum motion angle was 35° [47], which can verify our conclusion. Thus, the Wrist Radia Deviation can be widened to 40° so that the eigenvalues obtained from the experiment can be included. Even so, the measured mean value of Wrist Radia Deviation we get has reached the maximum value in theory. This further shows that the obvious force of gesture movement to extreme states is a special feeling, which can promote the formation of a characteristic state.

Among the ten motion primitive units, the wrist radial deviation and wrist ulnar deviation correspond to two relative motion directions of the same motion plane of the same joint, and the representative motion eigenvalue of their first semantic state were both 0°, which correspond to the same gesture. Therefore, the two motion primitive units can be combined into wrist deviation, and the ulnar side can be designated as the positive direction of the motion angle. Similarly, wrist flexion and wrist extension can also be combined into a wrist bending motion primitive unit, taking the flexion of the wrist as the positive direction of the movement angle.

### 3.4. Experiment 4: Empirical Experiment on State Division of Motion Primitive Units Based on Leap Motion

Through the above three experiments, the number of semantic states that could be conveyed by each motion primitive and the range of angle motion corresponding to each state were defined. Based on the abovementioned semantic state division, the gesture coding system could be improved and further applied to gesture interaction design and recognition. Nevertheless, a problem still exists in empirical applications. Due to the measurement deviation, there is a discrepancy between the gesture value measured by the gesture capture device and the actual gesture value executed by the user. Therefore, in empirical applications, the angle boundary corresponding to the motion state of each motion primitive should be adjusted according to the error characteristics of the gesture capture device. At the same time, the recognition algorithm should also be designed and improved according to the needs of the semantic expression of each motion primitive unit of the hand. In this experiment, taking Leap Motion as an example, the improvement suggestions of gesture capture algorithm were given by analyzing the deviation of the measurement system, and the angle boundary values of the motion state of each motion primitive unit were given by mapping between the measured and the actual hand value.

#### 3.4.1. Participants and Apparatus

A total of 10 participants (5 males and 5 females) between the age of 19 and 25 years (M = 22.6, SD = 1.560) were recruited from Southeast University for this experiment. Participants in this experiment were skilled user of Leap Motion. Moreover all participants were right-handed and performed the gestures with their right hands. Before the experiment, the participants read the experimental instructions and signed the informed consent form.

**Figure 13:**
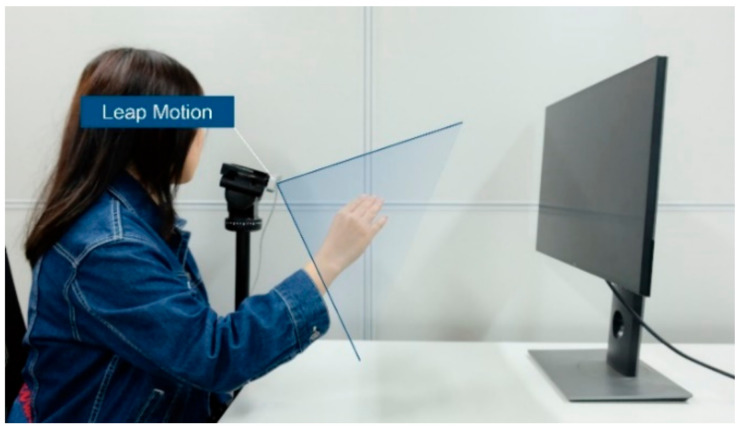
Schematic diagram of measurement using Leap Motion.

The computer host and experimental environment used in this experiment were the same as those in Experiments 1−3. An anthropometer was used to measure the actual value of the motion angle of the motion primitive units. Besides, as shown in Figure 13, a Leap Motion was used to capture and measure gestures. Leap Motion provided a Unity plug-in and thus enabled the mapping of real hand movements to virtual hand movements; i.e., participants could see the hand pose captured by Leap Motion on the screen. To enable participants to observe both the virtual hand and the real hand to balance the measurements of Leap Motion and the anthropometer, the Leap Motion was placed on a stand to simulate the use of VR mode.

#### 3.4.2. Task, Procedure and Experimental Design

**Figure 14:**
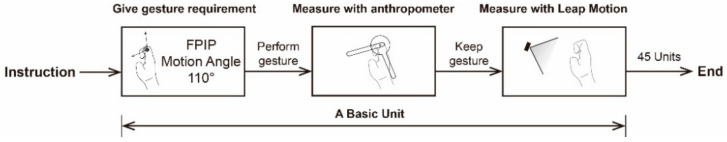
Experimental flowchart of practical experiment on state division of motion primitive units based on Leap Motion.

The flowchart of an experimental unit is shown in Figure 14. Moreover, there are three main stages included in a unit such as giving motion requirements, measurement based on ruler measurement, and measurement based on Leap Motion.

**Figure 15:**
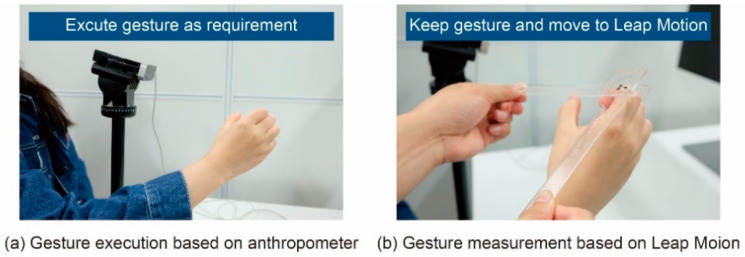
Schematic diagram of the experimental scene.

Firstly, the participants were asked to execute a gesture at the required angle of movement for a particular motor primitive. Then, as shown in Figure 15a, the anthropometer was used to assist the participants in adjusting the joint posture until the angle of a certain motion primitive executed by the participant were as required. Next, as shown in Figure 15b, the participants were asked to keep the gesture unchanged and move their right hand into the capture range of Leap Motion, and the gesture captured by Leap Motion was displayed on the monitor. Finally, once the gesture was captured by Leap Motion steadily, the participant was asked to press the space bar with their left hand to record the Leap Motion measurement data at that time.

As shown in Table 3, all the motion primitive units with semantic states were given five requirement angles, which were 0%, 25%, 50%, 75%, and 100% of the maximum range of motion.

#### 3.4.3. Results

From the above experiments, the results of the mean and SD (standard deviation) of the Leap Motion measurement values for each requirement angle of each motion primitive for ten subjects are shown in Table 4. Except for the measurement result (SD = 16.1) of finger PIP flexion at 55°, the standard deviations of the other measurement values are all within 12°, which is consistent with the measurement results obtained in the relevant literature [45].

Since the motion range of each motion primitive unit varied widely from 30 to 110 degrees, Equation (1) was used to normalize the average measurement error to better characterize the deviation between the measured results and the actual value.

(1) $$\mathrm{Normalized}\;\mathrm{Measure}\;\mathrm{Error}=\frac{\mathrm{Abs}\left(\mathrm{Measurement}\;\mathrm{Error}\right)}{\mathrm{Motion}\;\mathrm{Range}\;\mathrm{of}\;\mathrm{Motion}\;\mathrm{Unit}}$$

**Figure 16:**
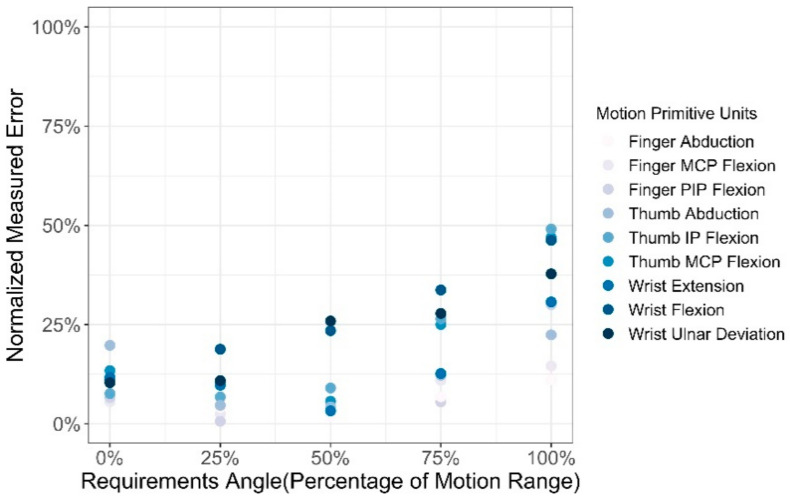
The normalized measurement error graph of each motion primitive.

Based on the above normalization formula, as shown in Figure 16, where the ratio of the motion angle to the motion range of the primitive units is used as the abscissa and the normalized data of the measurement error value is used as the ordinate, the normalized errors of all measurement data are displayed. As the motion angle of each motion primitive unit increases, the measurement error also increases gradually. When the motion angle reaches 80% of the maximum value, the normalized measurement error value of some motion primitive units exceeded 0.3, which means that the system deviation is large.

**Figure 17:**
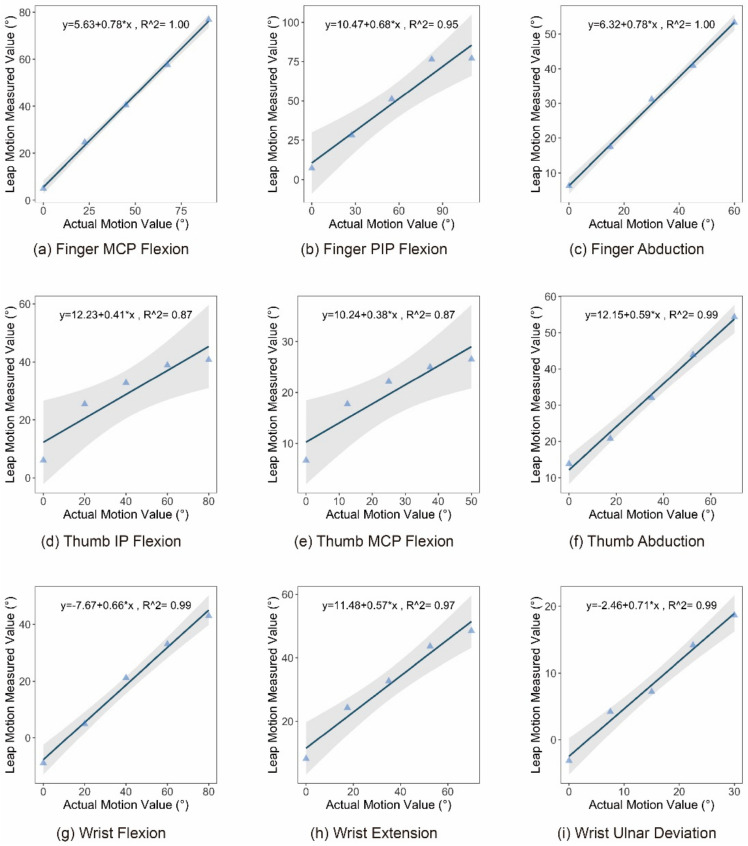
The linear fit graph between Leap Motion measured value and actual value for each hand motion primitive unit.

Further, to construct the mapping relationship between the Leap Motion measured and actual values, for each motion primitive unit, the actual value was taken as the abscissa, and the Leap Motion measurement value was taken as the ordinate. The results of linear fitting between the measured and actual values are shown in Figure 17.

#### 3.4.4. Discussion

Based on the above experimental results, it could be concluded that the Leap Motion measurement results have high stability, but there was a certain systematic deviation between the measured value and the actual value. According to the results shown in Figure 16, as the motion angle of the motion primitive increases, the deviation value increases. As discussed in Experiment 3, users tended to convey semantics through hand gestures that approach the limit state, but at this time, the data obtained from Leap Motion had a large deviation. Therefore, it is not recommended to use the measurement data from Leap Motion unadjusted.

According to a patent issued by Leap Motion [48], the measurement error may be caused by the constraints on the gesture preset in the capture algorithm. Moreover, the possible poor results of Leap Motion image edge extraction due to the lack of color information of the camera and the possible lack of training data under extreme motion conditions will also exacerbate the generation of errors. In summary, the algorithm can be adjusted according to the above reasons in order to obtain better gesture and pose capturing results.

However, at the current stage, before the measurement error is fixed, the mapping and conversion between Leap Motion measurement data and actual data are still necessary to help establish a good gesture recognition and gesture interaction experience. According to the linear fitting results of the measured and actual values obtained in Figure 17 (r^2^ are above 0.85, showing high linearity), the mapping formula between the two types of values is shown in Equation (2).

(2) $$\{\begin{array}{c} Finger\_MCP\_Flexion\_Leap=5.63+0.78*Finger\_MCP\_Flexion\_Actual\\ Finger\_PIP\_Flexion\_Leap=10.47+0.68*Finger\_PIP\_Flexion\_Actual\\ Finger\_Abduction\_Leap=6.32+0.78*Finger\_Abduction\_Actual\\ Thumb\_IP\_Leap=12.23+0.41*Thumb\_IP\_Actual\\ Thumb\_MCP\_Leap=10.24+0.38*Thumb\_MCP\_Actual\\ Thumb\_Abduction\_Leap=12.15+0.59*Thumb\_Abduction\_Actual\\ Wrist\_FLexion\_Leap=-7.67+0.66*Wrist\_FLexion\_Actual\\ Wrist\_Extension\_Leap=11.48+0.57*Wrist\_Extension\_Actual\\ Wrist\_Deviation\_Leap=-2.46+0.71*Wrist\_Deviation\_Actual \end{array}$$

## 4. Results

Based on the discussion at the end of Experiment 3, and combining the experimental results of the three experiments, the number of semantic states of the hand motion primitive units and the boundary value between each state could be obtained as shown in Table 5.

This result was consistent with the visual semantic perception of gestures by gesture observers and the semantic expression of gestures by the gesture executors. Due to differences in the range of joint motion of different people, in the interval division of the semantic state, the maximum and minimum motion angles of the motion primitive units were not set. Therefore, the program judgment error caused by the movement angle beyond the specified interval could be avoided.

Furthermore, based on the discussion results of Experiment 4, the boundary values of each motion state of each motion element in Table 5 were brought into the corresponding real-value Leap Motion measurement value mapping equation, and the Leap Motion-based gesture states classification result was obtained, as shown in Table 6. After empirical verification, this classification could be well applied in Leap Motion–based gesture state recognition.

## 5. General Discussion

Gesture is a kind of symbolic language, and it needs the consistency of meaning understanding within groups to realize the effective transmission of semantics. The three experiments described in this paper indicated a high consistency between the motion semantic expression and the visual perception of the meaning of hand movement primitives, which confirms the human mirror mechanism. From the results of Experiment 1 and Experiment 2, people’s perception of the meaning of the motion primitive units that constitute the hand gesture was limited and discrete. Aside from finger MCP flexion and PIP flexion, which can express three symbolic meanings, and wrist radial deviation, which can only express one symbolic meaning, all the other motion primitive units can express two symbolic meanings. From the results of Experiment 3, people’s reports on the state of motion primitive units directly verified the discrete meaning expression. In addition, people had high consistency in the judgement of the number of states of the semantic expression of motion primitive units and the execution of each state, which reflects a relatively high group consistency in peoples’ understanding of the meaning expression of motion primitive units.

Through the discussion of the three experimental results, we have merged the motion primitive units, which were from the same joint and had the same degree of freedom. We determined eight motion primitive units: finger MCP flexion, finger PIP flexion, finger abduction, thumb MCP flexion, thumb IP flexion, thumb abduction, wrist bending, and wrist deviation. Finally, the quantitative classification results of the semantic state of each motion primitive unit that were consistent within groups and conformed to human expression and perception were obtained and shown in Table 5. The number of joint semantic states of each motion primitive unit is between 2 and 3 and has a strong correlation with the motion amplitude of the motion primitive unit. The motion amplitude of the motion primitive unit with the number of states of 3 is greater than or equal to 90°. Except for the thumb MCP flexion, the characteristic motion angles of the first state of the other motion primitive units were all 0°, showing a high consistency. However, since the thumb MCP flexion has a particular angle in the extended state due to its bone structure even without external force intervention, it showed a characteristic value of 5°.

Based on Leap Motion, an application example of the above semantic state classification was introduced in Experiment 4. The discussion of the measurement error characteristics reveals the defect of Leap Motion; that is, it cannot effectively capture large motion angles. Since people tend to use extreme gesture states to convey semantics, the bad experience of gesture recognition and gesture interaction may be caused by that capture defect. Therefore, some algorithm-tuning suggestions can be used to improve gesture capture, including adjusting the constraints on gesture states, increasing the training data in extreme states, and adding RGB image capture. In addition, to make our gesture state classification data can be used based on the existing system, the gesture state classification of Leap Motion was also given based on the mapping relationship between the measured and actual values. The above analysis and mapping methods have some generality and can be extended to other gesture capture devices.

Discrete classification and representation of hand gestures with universal group consistency can be used as the basis of an effective gesture coding system. The discussion of the classification of each joint motion state in the existing sign language coding system is function oriented. Because the current sign language gestures are clear, when formulating the coding rules, the sign language gesture set is regarded as the target, and classification rules are formulated according to the expression needs of sign language gestures. The state classification of each motion primitive unit described in this article is considered from the perspective of semantic transmission and proposed based on the results of psychophysical experiments focusing on the description of the complete semantic expression space of gestures. Discretized hand gesture expression provides basic coding space for gestures. The gestures contained in this space exist in the continuous gesture motion space in the form of discrete points, representing all gestures with independent semantics. This means that the classification results described in this article are more objective and more systematic, and have a wider range of applications, especially in the field of natural interactive gestures. They provide a basic guide to the construction of a reasonable and complete interactive gesture coding system. The establishment of a perfect and objective gesture coding system can also further promote the formation of gesture-related standards in various fields and the construction of digital gesture resource libraries.

In previous studies on gesture coding and gesture representation [33,34,35], the semantic state of gestures was always customized based on the objective needs of the application scenarios. That was a reverse process. For example, the sign language coding system SignWriting divides the flexion of finger PIP into three basic states: straight, bend, and curve [3]. The result of the division is that these three states were needed in sign language expression, and these three states are also sufficient to cover all sign language actions. On the contrary, in our research, the division of semantic states was based on experiments of human subjective perception and expression of gestures, which was a forward process. Although we followed a completely different method from previous studies, the results we obtained showed strong consistency. In the sign language coding systems HamNoSys [33] and SignWriting [35], the flexion of the finger PIP and the finger MCP includes three states; the flexion of the thumb joints and the abduction between the fingers include two states. That is consistent with the results obtained in our research, and it also illustrates the usability of our results to a certain extent.

The semantic state classification results of the motion primitive units proposed in this paper not only determined the number of semantic states but also provided the corresponding boundaries and typical gestures of each state. Current findings lay the foundation for building bridges between human expression, perception, and computer recognition and output. The computer recognizes the position of the human hand joints to obtain data information, while the human expresses internal meaning. A clear quantitative standard can establish a mapping relationship between the two to support the presentation of gestures by the computer and the recognition of gestures. The result gives the movement direction, amplitude range, and typical motion angle value corresponding to each semantic state of each movement primitive unit. The typical angle values of the semantic states can be used to drive a virtual model to provide a basis for the storage and animation of gestures. Moreover, the angular range of each semantic state can provide a basis for gesture recognition. Mapping the gestures captured by the computer into a specific limited semantic space can greatly improve the efficiency of computer storage and recognition.

## 6. Conclusions

This study conducted an experimental analysis of the meaning transmission state of gesture motion primitive units from two levels, namely meaning perception from the observer’s perspective and meaning expression from the executor’s perspective, and combined the meaning expression of gesture semantics with the angle of joint movement. The radial curvature of the wrist joint was considered to be able to express only one state. Gesture motion primitive units such as thumb IP flexion and thumb MCP flexion, thumb TMC abduction and finger MCP abduction, and wrist bending and wrist deviation were divided into two semantic states, while finger PIP flexion and finger MCP flexion were divided into three semantic states (Table 5). The motion angle range of the motion primitive unit corresponding to each state and the most representative characteristic angle were also determined. Furthermore, the measurement errors in hand motion capture were experimentally measured for Leap Motion and possible suggestions for algorithm improvement were given. Moreover, based on the measurement results of Leap Motion, the mapping relationship between the measured values and the actual motion values of the gesture executor was fitted for the current stage of application. Moreover, the semantic state classification of each gesture motion primitive unit for Leap Motion measurement values was also given (Table 6).

The continuous and infinite gesture motion space was simplified into discrete and limited gesture semantic expression spaces, with a clear boundary between each discrete state, which can help establish an objective mapping relationship between the data information obtained by the computer and the human expression. The coding of gestures can be carried out on this basis. It satisfies the objectivity requirements of gesture communication between people, especially gesture researchers and designers, and is convenient for computer storage and interpretation, which will further promote gestures and the formation of gesture standards and the construction of a digital gesture library in different fields. In addition, the semantic expression space clarifies the meaning expression ability and expression characteristics of each motion primitive unit, particularly significant for guiding the development of gesture recognition technology.

In the future, the gesture semantic expression space described in this article can be further expanded. The semantic expression of gestures considered in this paper only included the static motion state of each motion primitive unit, which corresponded to the pose state of the hand. However, the expression of gestures also includes changes in the overall position of the hand, such as the general movement of the hand position in a waving action. The semantic state conveyed by these overall hand movements should be considered in future work.

## Figures and Tables

**Table 1:** The active range of motion of the wrist and joints of hand.

Joint	Active Range of Motion (°)			
	Flexion-Extension		Abduction–Adduction	
	Flexion (°)	Extension (°)	Abduction (Finger)/ Ulnar Deviation (Wrist) (°)	Adduction (Finger)/ Radial Deviation (Wrist) (°)
Finger MCP	0–90	0–20	0–60	0
Finger PIP	0–110	0	0	0
Finger DIP	0–70	0	0	0
Thumb MCP	0–50	0	0	0
Thumb IP	0–80	0–10	0	0
Thumb TMC	0–15	0–20	0–70	0
Wrist	0–80	0–70	0–30	0–20

**Table 2:** Stimulation configuration for Experiment 1.

Joint	Motion Form	Standard Stimulus Parameters (°)	Contrast Gesture Value Interval (°)	Number of Contrast Gestures
Finger MCP	Flexion	0/90	10	10
	Abduction	0/60	10	7
Finger PIP	Flexion	0/110	10	12
Thumb MCP	Flexion	0/50	10	6
Thumb IP	Flexion	0/80	10	9
Thumb TMC	Abduction	0/70	10	8
Wrist	Flexion	0/80	10	9
	Extension	0/70	10	8
	Radial Deviation	0/20	5	5
	Ulnar Deviation	0/30	5	7

**Table 3:** Requirement angles configuration of motion primitive units for Experiment 1.

Motion Unit	Actual Value	Measured Value (SD)	Motion Unit	Actual Value	Measured Value (SD)	Motion Unit	Actual Value	Measured Value (SD)
Finger MCP Flexion	0	5.0 (3.5)	Thumb IP Flexion	0	6.1 (5.4)	Wrist Flexion	0	−8.8 (9.6)
Finger MCP Flexion	22.5	24.7 (5.7)	Thumb IP Flexion	20	25.4 (10.2)	Wrist Flexion	20	5.0 (8.9)
Finger MCP Flexion	45	40.4 (7.7)	Thumb IP Flexion	40	32.8 (8.9)	Wrist Flexion	40	21.2 (11.4)
Finger MCP Flexion	67.5	57.6 (4.5)	Thumb IP Flexion	60	38.9 (7.2)	Wrist Flexion	60	33.0 (11.1)
Finger MCP Flexion	90	76.9 (9.9)	Thumb IP Flexion	80	40.7 (7.6)	Wrist Flexion	80	43.0 (9.2)
Finger PIP Flexion	0	7.3 (4.7)	Finger Abduction	0	−6.3 (3.5)	Wrist extension	0	8.2 (10.5)
Finger PIP Flexion	27.5	28.2 (9.3)	Finger Abduction	15	−17.5 (7.9)	Wrist extension	17.5	24.3 (7.1)
Finger PIP Flexion	55	51.1 (16.1)	Finger Abduction	30	−31.2 (11.0)	Wrist extension	35	32.7 (8.9)
Finger PIP Flexion	82.5	76.4 (8.1)	Finger Abduction	45	−40.9 (9.1)	Wrist extension	52.5	43.6 (8.2)
Finger PIP Flexion	110	77.0 (11.8)	Finger Abduction	60	−53.3 (6.8)	Wrist extension	70	48.5 (11.2)
Thumb MCP Flexion	0	6.7 (6.4)	Thumb Abduction	0	13.8 (6.6)	Wrist Deviation	0	−3.1 (3.3)
Thumb MCP Flexion	12.5	17.7 (5.2)	Thumb Abduction	17.5	20.8 (3.9)	Wrist Deviation	7.5	4.2 (4.4)
Thumb MCP Flexion	25	22.2 (3.7)	Thumb Abduction	35	32.0 (5.7)	Wrist Deviation	15	7.2 (4.6)
Thumb MCP Flexion	37.5	25.0 (4.6)	Thumb Abduction	52.5	43.9 (8.6)	Wrist Deviation	22.5	14.2 (5.8)
Thumb MCP Flexion	50	26.5 (4.1)	Thumb Abduction	70	54.3 (7.6)	Wrist Deviation	30	18.7 (4.4)

**Table 4:** Mean and standard deviation of Leap Motion measurement results.

Joint	Motion Form	Range of Motion (°)	Requirement Angle 1 (°)	Requirement Angle 2 (°)	Requirement Angle 3 (°)	Requirement Angle 4 (°)	Requirement Angle 5 (°)
Finger MCP	Flexion	0–90	0	22.5	45	67.5	90
	Abduction	0–60	0	15	30	45	60
Finger PIP	Flexion	0–110	0	27.5	55	82.5	110
Thumb MCP	Flexion	0–50	0	12.5	25	37.5	50
Thumb IP	Flexion	0–80	0	20	40	60	80
Thumb TMC	Abduction	0–70	0	17.5	35	52.5	70
Wrist	Flexion	0–80	0	20	40	60	80
	Extension	0–70	0	17.5	35	52.5	70
	Deviation	0–30	0	7.5	15	22.5	30

**Table 5:** Semantic state classification results of each hand motion primitive unit for the real world.

Joint	Motion Form	Number of Semantic States	Angle Range of State 1 (°)	Expected Value of State 1 (°)	Angle Range of State 2 (°)	Expected Value of State 2 (°)	Angle Range of State 3 (°)	Expected Value of State 3 (°)
Finger MCP	Flexion	3	0–22.4	0	22.4–60.0	40	70.4–90.0	74
Finger PIP	Flexion	3	0–30.1	0	30.1–80.4	51	80.4–110.0	91
Finger MCP	Abduction	2	0–23.4	0	23.4–60.0	44	/	/
Thumb MCP	Flexion	2	0–24.3	5	24.3–50.0	49	/	/
Thumb IP	Flexion	2	0–29.4	0	29.4–80.0	74	/	/
Thumb TMC	Abduction	2	0–27.0	0	27.0–70.0	53	/	/
Wrist	Flexion/ Extension	3	−70.0–−34.1	−46	−34.1–37.2	0	37.2–80	49
Wrist	Deviation	2	−20–11.1	0	11.1–40	38	/	/

**Table 6:** Semantic state classification results of each hand motion primitive unit for Leap Motion digital world.

Joint	Motion Form	Number of Semantic States	Angle Range of State 1 (°)	Expected Value of State 1 (°)	Angle Range of State 2 (°)	Expected Value of State 2 (°)	Angle Range of State 3 (°)	Expected Value of State 3 (°)
Finger MCP	Flexion	3	<22.4	0	22.4–70.4	40	70.4<	74
Finger PIP	Flexion	3	<30.1	0	30.1–80.4	51	80.4<	91
Finger MCP	Abduction	2	<23.4	0	23.4<	44	/	/
Thumb MCP	Flexion	2	<24.3	5	24.3<	49	/	/
Thumb IP	Flexion	2	<29.4	0	29.4<	74	/	/
Thumb TMC	Abduction	2	<27.0	0	27.0<	53	/	/
Wrist	Flexion/ Extension	3	<−34.1	−46	−34.1–37.2	0	37.2<	49
Wrist	Deviation	2	<11.1	0	11.1<	38	/	/

## Data Availability

The experiment data and data-handler programs can be found online at https://github.com/LELEJIA/The-Results-of-Research-on-Discrete-Semantics-in-Continuous-Hand-Joint-Movement-Based-on-Perception (accessed on 20 April 2021).
